# The Gap Between Perceived and Objective Diet Quality in Patients with Lynch Syndrome

**DOI:** 10.3390/nu18193284

**Published:** 2026-10-06

**Authors:** Jessica Fitzpatrick, Daniel Clayton-Chubb, Sarah Melton, Douglas Tjandra, Jadon Karp, Daniel Zhu, Kate Dunbar, Gregor Brown, Alex Boussioutas

**Affiliations:** 1 Department of Gastroenterology, School of Translational Medicine, Monash University, Clayton, VIC 3800, Australiaalexb@alfred.org.au (A.B.); 2 Department of Gastroenterology, Alfred Hospital, Melbourne, VIC 3004, Australiakate.dunbar@alfred.org.au (K.D.);; 3 Clinical and Translational Epidemiology Unit, Massachusetts General Hospital, Boston, MA 02114, USA

**Keywords:** Lynch, dietary quality, mediterranean diet, cancer worry score, colorectal cancer

## Abstract

**Background and Aims**: Lynch syndrome (LS) confers a substantially elevated lifetime risk of colorectal cancer (CRC). While enhanced endoscopic surveillance and aspirin chemoprevention are the cornerstones of management, evidence suggests modifiable lifestyle factors like diet may help mitigate or modify hereditary cancer risk. Because specific LS dietary guidelines are lacking, general CRC prevention recommendations are often substituted. Therefore, the aim of this study was to conduct a detailed and comprehensive examination of dietary habits, cancer-related dietary beliefs, and overall cancer-related worry in individuals with Lynch syndrome who attend a Gastrointestinal (GI) Risk Management Clinic. **Methods**: A cross-sectional study was conducted using data collected between June 2025 and June 2026 from individuals with a confirmed LS diagnosis attending a tertiary Gastrointestinal Risk Management Clinic in Melbourne, Australia. Participants completed electronic questionnaires capturing demographics, clinical history, a Food Frequency Questionnaire, the 14-item Mediterranean Diet Adherence Screener (MEDAS), and the Cancer Worry Scale (CWS). Self-reported dietary beliefs regarding the relationship between food and cancer risk were captured. Lifestyle behaviors were benchmarked against the Australian Bowel Cancer Risk Reduction Recommendations. The MEDAS was primarily analysed as a population-specific top-vs-bottom half (“better adherence”), and secondarily as a priori “good” adherence (MEDAS > 7). **Results**: A total of 94 LS patients completed the study (65% female, median age ~51). A striking paradox was observed: 73.3% of participants believed they maintained a healthy diet, yet the cohort demonstrated poor objective adherence to a Mediterranean pattern (median MEDAS: 5 [IQR 4, 7]; range 0–10), with only 14.9% achieving a “good adherence” score (>7). Cancer worry was prevalent, with 60.9% having a CWS > 13, consistent with significant worry about future cancer, with no sex-based differences. However, higher cancer worry (CWS > 11: aOR 1.59, 95% CI 0.48–5.28) and a personal history of cancer (aOR 0.69, 95% CI 0.24–1.97) were not significantly associated with better dietary adherence. Aspirin use was a strong predictor of better Mediterranean diet adherence, yielding an aOR of 3.09 (95% CI: 1.10–8.68), likely reflecting improved overall engagement in health promotion. Only one participant (1.1%) met all objective bowel cancer prevention guidelines. Of the cohort, 37% intentionally restricted foods (primarily processed/red meats) and 21% intentionally included specific foods to prevent cancer; their primary information sources were doctors and the internet. **Conclusions**: A substantial gap exists between perceived diet quality and objective adherence to evidence-based cancer prevention recommendations among individuals with Lynch syndrome. High levels of cancer-related worry and a willingness to modify dietary choices do not automatically translate into optimal nutrition. These findings highlight a critical need for interventional trials to provide LS patients with evidence-based dietary guidelines to reduce their lifetime risk of CRC that are tailored to this highly motivated but clinically underserved population.

## 1. Introduction

Lynch syndrome (LS) is the most common cause of hereditary colorectal cancer (CRC), arising from pathogenic germline variants in the DNA mismatch repair genes (MLH1, MSH2, MSH6, PMS2) or deletion of the EPCAM gene [1]. Estimated to affect approximately 1 in 440 individuals yet accounting for as many as 5% of all CRC diagnoses, LS confers substantially elevated lifetime risks of CRC, endometrial cancer, and malignancies at several other sites [1]. Management of CRC risk in LS focuses primarily on aspirin chemoprevention and enhanced endoscopic surveillance, and while this remains the cornerstone of care, interest in modifiable lifestyle factors as a potential adjunct for cancer risk reduction has grown considerably in recent years [2,3].

This interest has arisen in part because of the variable penetrance of CRC in LS. Despite sharing the same underlying genetic predisposition, CRC rates vary considerably among individuals with LS, even among those harboring variants in the same MMR gene [2,4]. This variability in cancer expression points to the likely contribution of environmental and lifestyle factors in modulating risk above and beyond an individual’s underlying genetic susceptibility [5,6,7]. As such, understanding which modifiable factors are relevant in LS, and the extent to which individuals with the syndrome engage with them, is of considerable clinical importance.

Diet is among the most extensively studied environmental determinants of CRC risk in the general population. Higher intakes of red and processed meat have been consistently associated with increased CRC risk, while dietary fiber and adherence to a Mediterranean dietary pattern have each been associated with meaningful reductions in both sporadic CRC and colorectal adenoma incidence [4,8,9,10,11]. Within LS, specifically, obesity has been associated with the development of colorectal tumors and adenomas [12] while dietary patterns validated for CRC detection have not yet been well explored [13,14,15]. Considering the variable penetrance of colorectal cancer in Lynch syndrome, investigating modifiable dietary patterns offers a valuable opportunity to identify novel strategies for risk mitigation.

Given their awareness of substantially elevated cancer risk, it has been hypothesized that individuals with LS may adopt healthier dietary and lifestyle behaviors than the general population. The available evidence, however, does not consistently support this assumption. While individuals with LS and those with other non-LS hereditary CRC risks do appear to endorse beliefs that diet can influence cancer development [16], their actual dietary behaviors do not reflect this awareness [17]. Poor adherence to cancer prevention guidelines, high rates of red meat consumption, and inadequate intake of fruit, vegetables, and fiber have been documented consistently across different LS cohorts [5,18,19]. Importantly, however, dietary patterns and dietary beliefs in LS have largely been examined in isolation and across separate study populations, limiting the ability to draw integrated conclusions about what this group believes, what they eat, and how this compares to evidence-based cancer prevention recommendations.

Therefore, the aim of this study was to conduct a detailed and comprehensive examination of dietary habits, cancer-related dietary beliefs, and overall cancer-related worry in individuals with Lynch syndrome who attend a Gastrointestinal (GI) Risk Management Clinic. Dietary intake was assessed using a food frequency questionnaire, with additional characterization of Mediterranean diet adherence, dietary supplement use, and physical activity behaviors. Self-reported dietary beliefs regarding the relationship between food and cancer risk were captured, and the cancer worry scale (as a repurposed marker for fear of cancer occurrence) was calculated. Collectively, dietary behaviors were compared against established general bowel cancer prevention recommendations to characterize the general adherence to risk reduction strategies, even if their effectiveness has not been specifically researched in an LS population.

## 2. Materials and Methods

### 2.1. Study Design and Setting

A cross-sectional study was conducted using data extracted from a prospectively maintained database at the Gastrointestinal Risk Management Clinic (GIRMC) within the Alfred Care Group, a tertiary health service located in Melbourne, Australia. Data collection spanned from June 2025 to June 2026.

### 2.2. Participant Recruitment and Eligibility

**Figure 1:**
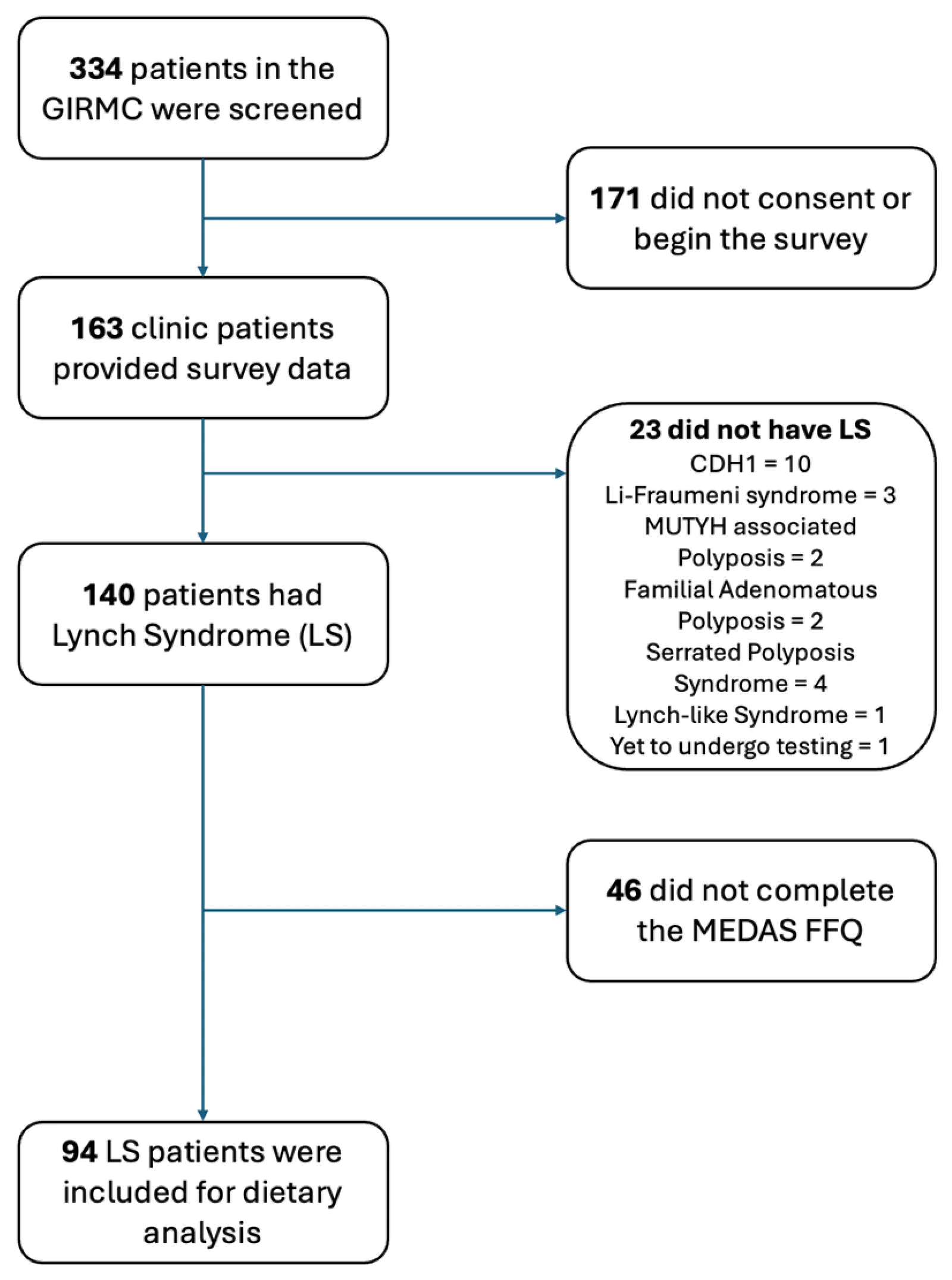
Patient flow.

Eligible participants included individuals with a confirmed diagnosis of LS who were referred to and attended the GIRMC during the study period. Upon referral, patients were invited to participate in the study; those who formally opted in, provided informed consent, and subsequently adequately completed the comprehensive intake and lifestyle questionnaire administered electronically via REDCap (Research Electronic Data Capture) were included (Figure 1).

### 2.3. Data Collection and Variables

Demographic and clinical characteristics were captured at baseline, including age, sex, smoking status, country of birth, personal history of cancer diagnosis, family history of cancer, and LS genotype from their electronic medical record. Participants self-reported their highest level of education.

### 2.4. Lifestyle Questionnaire

Participants self-reported their height and weight, which were used to calculate Body Mass Index.

Nutritional intake and physical activity levels were evaluated via a comprehensive lifestyle survey containing the following validated instruments and metrics:

### 2.5. Dietary Intake and Component Analysis

Habitual dietary intake and adherence to specific nutritional patterns were evaluated using a comprehensive, self-administered food frequency questionnaire (FFQ) consisting of 40 targeted items. The questionnaire was designed to capture eating habits over the past week using semi-quantitative categorical response categories. These questions evaluated both daily and weekly consumption frequencies across core food groups, alongside specific culinary behaviors (e.g., preference for white over red meat, usage of sofrito, and the primary choice of culinary fats). Frequencies were converted to absolute metric intakes (grams or milliliters per day or week) based on standard portion sizing.

The FFQ was structured to isolate specific dietary components hypothesized to influence colorectal health. The questionnaire evaluated and differentiated between traditional fermented dairy (e.g., yogurt, kefir) and non-dairy fermented alternatives, alongside other distinct fermented foods and beverages such as kombucha, sauerkraut, and kimchi. It also separated whole grains, seeds, legumes, fruit, vegetables, and nuts into independent exposure categories, tracked plant-based milk and dairy substitutes, and explicitly isolated oily fish (e.g., salmon, mackerel, tuna, sardines) from general seafood intake. Refined beverage subtypes were analyzed by disaggregating sugar-sweetened beverages (e.g., standard soft drinks, energy drinks, cordial) from artificially sweetened diet drinks. Furthermore, the FFQ captured distinct subdivisions of ultra-processed foods through discrete evaluations of commercial sweet items (e.g., pastries, cakes, biscuits), savory convenience items (e.g., frozen or prepared meals), protein powders, and commercially available meal replacement or diet products. Finally, established oncogenic risk factors were accounted for by quantifying detailed categories of animal protein intake, explicitly separating processed meats from unprocessed red meats, and non-red wine alcohol from red wine.

### 2.6. Mediterranean Diet Adherence

Adherence to a Mediterranean-style diet was quantified using the validated 14-item Mediterranean Diet Adherence Score (MEDAS), which asks about dietary habits over a week [20]. To assess predictors of optimal nutrition, MEDAS scores were dichotomized into a binary outcome (with the top half reflecting population-specific “better” adherence), whereas a score greater than 7 was defined a priori as indicating “good adherence” [21] This approach was taken due to prior work showing relatively poor baseline Mediterranean diet adherence in Australians with established cardiovascular disease, where only 6.2% achieved a score ≥ 9 [22].

### 2.7. Physical Activity

Adherence to physical activity guidelines was assessed utilizing questions mapped to the Australian National Physical Activity Recommendations [23].

### 2.8. Dietary Attitudes and Perceptions

Participant perceptions of their own dietary habits were captured using a binary question regarding whether they felt their current diet was “healthy” or “unhealthy”. Additionally, specific dietary attitudes were evaluated, including targeted questions on the intentional inclusion or avoidance of specific foods or dietary supplements for the purpose of cancer prevention, alongside the primary sources from which participants gathered dietary information regarding food and cancer.

To evaluate lifestyle-associated cancer risk, participant data were analyzed and benchmarked against the objective criteria outlined in the Australian Bowel Cancer Risk Reduction Recommendations [24]. Adherence rates were calculated for individual dietary components (avoiding processed meat, having 400 g dairy products a day, having 90 g wholegrains a day, no more than 2 standard drinks of alcohol a day), physical activity thresholds (30 min of moderate physical activity a day), non-smoking recommendations and weight parameters (BMI between 18.5 kg/m^2^ and 25 kg/m^2^). The red meat recommendations were <455 g/week [25].

### 2.9. Cancer Worry Scale

Psychological burden related to oncological risk was measured using the Cancer Worry Scale (CWS) [26]. To thoroughly explore the impact of cancer-related anxiety, CWS scores were analyzed categorically using two distinct, established clinical cutoff points: low (CWS ≤ 11) vs. elevated worry (CWS > 11), which is used for screening, and low-to-moderate (CWS ≤ 13) vs. severe worry of cancer recurrence (CWS > 13) [26,27].

### 2.10. Genetic Risk Status

Patients were stratified by genetic risk status (high risk; MLH1/MSH2/EPCAM vs. low risk; MSH6/PMS2) to explore potential associations with dietary outcomes [28].

### 2.11. Statistical Analysis

All statistical analyses were performed using Stata 19.5 MP (College Station, TX, USA; StataCorp LLC). Descriptive statistics were used to summarize baseline cohort characteristics. Continuous variables were assessed for normality and are presented as means standard deviations or medians with interquartile ranges, as appropriate. Categorical variables are expressed as frequencies and percentages.

The association between continuous MEDAS scores and patient demographic characteristics was evaluated using Spearman’s rank correlation coefficient. To identify independent predictors of good Mediterranean diet adherence, multivariate logistic regression modeling was utilized. Covariates and independent factors, including demographic features, clinical history, and the distinct categorical brackets of the CWS, were entered into the multivariate models. Measures of association are reported as adjusted odds ratios accompanied by their corresponding 95% confidence intervals. Given the exploratory nature of this survey, no formal adjustments for multiple comparisons were made to avoid omitting potentially important associations (Type II error). Consequently, a two-sided *p*-value of <0.05 was considered statistically significant, and all findings should be interpreted as hypothesis-generating. No imputation was performed for missing variables.

## 3. Results

### 3.1. Baseline Characteristics & Demography

The dietary questionnaire was completed by 94 of 140 surveyed individuals with Lynch syndrome (67.0% response rate; 65% female) (Figure 1). There were no significant differences in key baseline characteristics between males and females (Table 1) apart from a greater median number of days of strength training per week for females (2 [0–4] vs. 0 [0–2], *p* = 0.03). Overall, 73.3% (66/90) believed they had a healthy diet despite the cohort having a relatively poor overall adherence to a Mediterranean dietary pattern (median MEDAS 5 [IQR 4, 7]). Only 14 participants (14.9%) had good adherence to a Mediterranean dietary pattern (MEDAS score > 7).

### 3.2. MEDAS Score, Aspirin Use and Demographics

There was no significant relationship between overall MEDAS score and age (Spearman’s rho 0.14, *p* = 0.20) or sex (*p* = 0.11). Similarly, there was no relationship between age (OR 1.02 [95% CI 0.99–1.06]; OR 1.04 [95% CI 0.99–1.08]) or female sex (OR 1.82 [95% CI 0.77–4.29]; OR 1.42 [95% CI 0.41–4.94]) and a “better” or “good” MEDAS, respectively. When evaluating the “better” MEDAS binary cut-point, there was no age- and sex-adjusted relationship observed between genetic risk status (high-risk; MLH1/MSH2/EPCAM vs. low risk; MSH6/PMS2) (aOR 0.68, 95% CI 0.28–1.67). While smoking was not significantly associated with better MEDAS adherence (aOR 1.18, 95% CI: 0.18–7.74), this analysis was constrained by a low prevalence of smokers in the cohort (5.7% current smokers). No participants with a MEDAS > 7 were current smokers.

Aspirin use was a strong predictor of better Mediterranean diet adherence, yielding an aOR of 3.09 (95% CI: 1.10–8.68). This association was not attenuated when additionally adjusting for BMI, smoking status, and family history of cancer (aOR 4.34, 95% CI: 1.22–15.41). This most likely relates to a general increase in health-conscious behavior driving both increasing aspirin utilization and concomitant improved MEDAS values.

There was a significant negative relationship between MEDAS score and BMI (Spearman’s rho −0.27, *p* = 0.01). Similarly, when assessed continuously, higher BMI was significantly associated with a decreased probability of high MEDAS adherence, with an age- and sex-adjusted odds reduction of 11% for every one-unit increase in BMI (aOR 0.89, 95% CI: 0.81–0.97).

Dietary motivations also significantly influenced MEDAS adherence; after adjusting for age and sex, individuals who did not actively restrict their diets to avoid cancer had significantly lower odds of achieving a high MEDAS score (aOR 0.33, 95% CI: 0.13–0.80). A similar, though non-significant, trend toward lower MEDAS scores was observed among participants who did not deliberately add healthy foods to their diet (aOR 0.39, 95% CI: 0.13–1.16).

### 3.3. MEDAS Components

When considering the potential contributors to the MEDAS score, most participants consumed olive oil as their main culinary fat, consumed few servings of sweet or carbonated beverages, and had 2 or more servings of vegetables per day (Table 2). However, very few had adequate legumes (3 serves per week or more), ate adequate seafood, consumed adequate amounts of olive oil, or consumed enough nuts (Table 2).

### 3.4. Adherence to Lifestyle Prevention Strategies for Colorectal Cancer

There were no male participants and one female participant (2%) who met all the Bowel Cancer Australia recommendations to reduce the risk of CRC (Table 3) [24,25]. The category with the lowest adherence rate for males was having a BMI < 25 kg/m^2^ (23%), and for females was eating adequate amounts of whole grains (24%). The only significant difference between males and females centered on processed meat intake, where only 50% of males consumed no processed meat, compared with 73% of females (*p* = 0.03). The vast majority of this LS cohort did not smoke, did not drink to excess, and exercised at least 30 min per day.

### 3.5. Cancer Worry, Diet, and Risk Status

Using the CWS, the majority of patients reported at least some concern around the development of cancer (CWS > 11) (Table 1). Similarly, 60.9% had a CWS > 13, consistent with significant worry about future cancer, with no sex-based differences. However, cancer worry was not significantly related to improved dietary quality, as defined by adherence to the MEDAS. Adjusted for age and sex, CWS > 11 was not associated with either a population-based “better” MEDAS score (aOR 1.59 [95% CI 0.48–5.28]) or a more rigorous MEDAS > 7 (aOR 0.42 [95% CI 0.11–1.65]). Similar results are seen using the more specific cut-off of CWS > 13 (aOR 0.83 [95% CI 0.35–1.97] and aOR 0.62 [95% CI 0.19–1.95], respectively). A self-reported personal history of cancer was also not related to a better MEDAS score (aOR 2.46 [95% CI 0.62–9.68]).

Genetic risk status (stratified as higher vs. lower MMR risk) did not demonstrate a significant relationship with standard CWS thresholds, including cut-offs at ≤11 vs. >11 (aOR 0.71, 95% CI: 0.20–2.48), and ≤13 vs. >13 (aOR 1.72, 95% CI: 0.67– 4.37). Genetic risk status was also not associated with aspirin use (aOR 0.66, 95% CI: 0.24–1.81). Similarly, a family history of cancer was not associated with either a better MEDAS score (aOR 1.06, 95% CI: 0.06–17.89) or CWS > 13 (aOR 1.87, 95% CI 0.11–31.87). Specifically having a first degree relative with a history of cancer, when adjusted for age- and sex, bore no statistically significant association with for the MEDAS binary cutpoint (aOR 1.52, 95% CI 0.58–3.98, *p* = 0.393), a CWS greater than 11 (aOR 1.32, 95% CI 0.36–4.91, *p* = 0.676), or a CWS greater than 13 (aOR 1.21, 95% CI: 0.46–3.17, *p* = 0.692).

### 3.6. Dietary Perceptions

**Figure 2:**
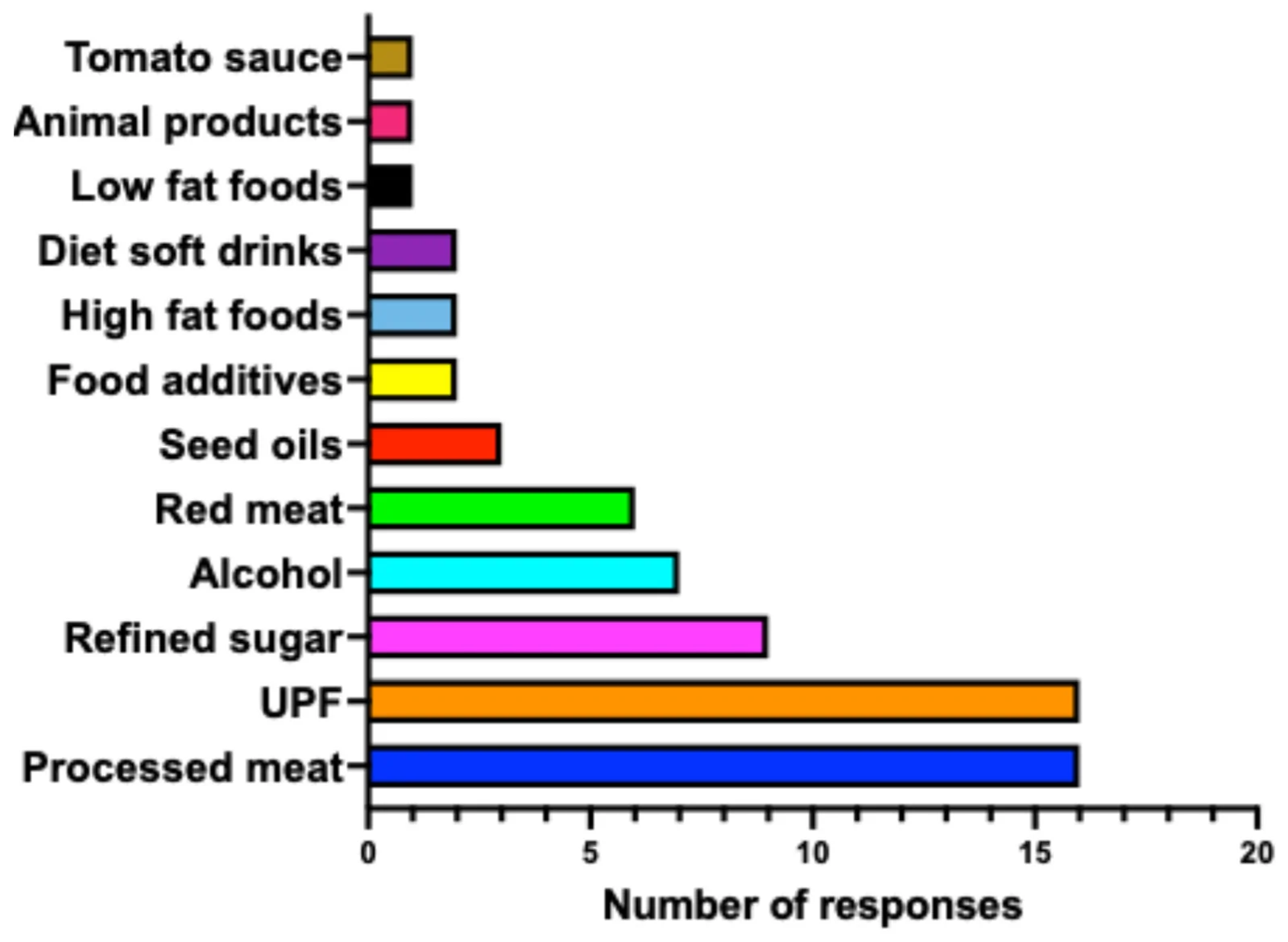
Responses to the question “Do you avoid any foods or ingredients to prevent the development of cancer?”

**Figure 3:**
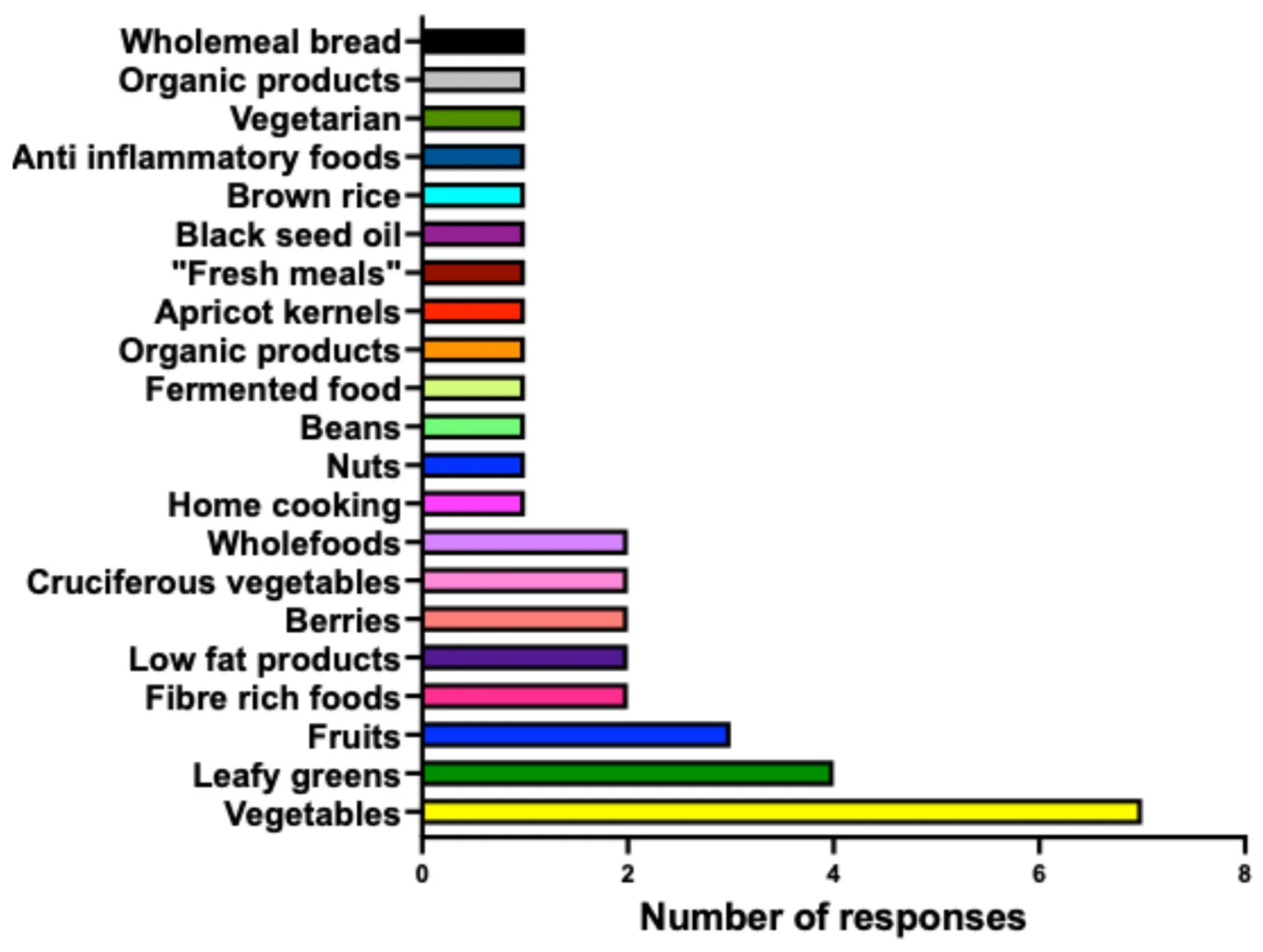
Responses to the question “Do you include any foods or ingredients to prevent the development of cancer?”.

**Figure 4:**
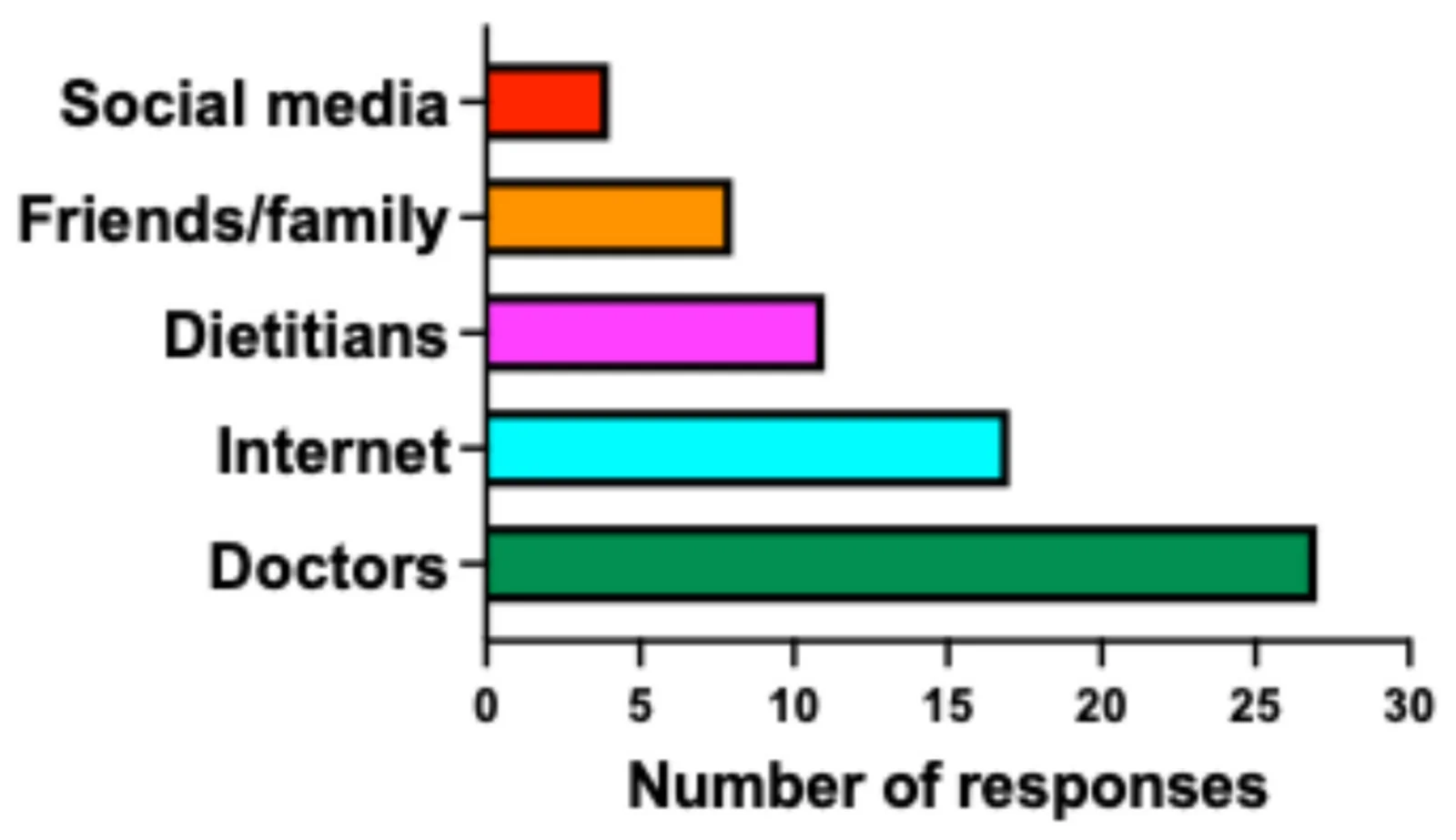
Responses to the question “Where did you find out information about restricting or including foods or ingredients?”.

35 respondents (37%) reported avoiding specific foods to prevent cancer, with the highest restriction including processed meat, ultra-processed food (UPF), and red meat (Figure 2). 20 respondents (21%) reported including foods to prevent cancer, with the most common responses including high-fiber foods such as vegetables, fruits, and nuts (Figure 3). There were no sex differences between men and women regarding foods they included or avoided to prevent the development of cancer. Only 5 participants reported taking supplements to reduce cancer risk which included Vitamin D (n = 2), omega 3s (n = 1), turmeric (n = 1), serrapeptase (n = 1), vitamins unspecified (n = 1), and psyllium (n = 1). Doctors (40%) and the internet (25%) were the most common places for participants to obtain information that recommended them to restrict or include dietary components to prevent bowel cancer (Figure 4).

## 4. Discussion

Our study reveals a stark paradox within the Lynch syndrome (LS) population, where dietary perceptions do not match adherence to healthy lifestyle patterns. While nearly three-quarters of our cohort (73.3%) believed they consumed a healthy diet, objective evaluation demonstrated poor overall adherence to a Mediterranean dietary pattern, evidenced by a median MEDAS score [20] of 5 out of 14. Similarly, when benchmarked against the Australian Bowel Cancer Risk Reduction Recommendations, only 1 participant met all the recommendations for general CRC risk reduction. Interestingly, a dichotomy emerged between dietary and non-dietary behaviors; participants demonstrated exceptionally high adherence to non-dietary lifestyle guidelines, with 90% to 96% identifying as non-smokers, 95% remained below the threshold of 14 standard drinks per week, and 78% to 90% achieved the target of 30 min of daily aerobic exercise, providing some evidence that targeted education focusing on beneficial dietary habits may be required for risk management in this population in the future.

### 4.1. Dietary Quality Adherence and Lynch Syndrome

These findings directly reflect a growing body of international literature showing that LS patients consistently struggle with dietary quality and guideline adherence. Despite their high-risk status, LS patients consumed less fiber, fruits, and vegetables, and more red and processed meat than matched healthy controls [19]. Consistent with this overall poorer pattern of eating, they also had lower Mediterranean diet scores in a French cohort. Consistent with these findings, an international cohort of Lynch syndrome (LS) patients demonstrated poor compliance with the World Cancer Research Fund guidelines; the median adherence score was a mere 2.5 out of 7, with fewer than half of the participants meeting the criteria for almost all lifestyle recommendations [18]. Interestingly, in the latter study, only 16% met physical activity guidelines, markedly lower than our Australian cohort.

This behavioral gap highlights a clear psychological contradiction in LS: patients are deeply aware of their inherited cancer risk—indeed, our cohort are actively engaged in a high genetic risk cancer service and had a high survey response rate—yet their actual diets fail to meet broad cancer prevention guidelines. This shortfall is not necessarily driven by a lack of awareness, or even motivation. In fact, Palmquist et al. established that the vast majority of individuals undergoing genetic evaluation (83%) are highly ‘action-oriented,’ firmly believing that proactive dietary changes can successfully reduce their cancer risk [16]. This proactive mindset is heavily driven by knowledge of their genetic risk as well as a personal or family history of cancer. This high baseline motivation is further supported by the Health-4-Families pilot trial [29]. In their evaluation of a digital lifestyle intervention for hereditary cancer families, they observed exceptional recruitment success, with 86% of eligible high-risk individuals consenting to participate and 79% completing baseline testing. These findings strongly reinforce the idea that individuals carrying pathogenic mismatch repair (MMR) variants are highly motivated to participate in tailored lifestyle and dietary interventions. This emphasizes that awareness of cancer risk alone does not appear sufficient to drive behavior change. This has implications for hereditary cancer services and primary care management, where patients may receive extensive surveillance and risk counselling but comparatively less structured support for lifestyle modification.

Patients with LS are also worried about developing cancer: in our cohort, over 80% reported elevated cancer worry (CWS > 11), with more than 60% experiencing severe, clinically significant worry (CWS > 13). However, regression modeling revealed that neither high cancer worry nor a personal history of cancer was associated with better diet quality. Our findings confirm that this concern rarely translates into protective dietary behaviors in the absence of structured support and evidence-based recommendations. This has also been demonstrated in a Dutch cohort of LS, where it was shown that a personal diagnosis of CRC did not improve dietary patterns over time [17], despite awareness of the potential role of diet in general bowel cancer prevention [30]. Therefore, high levels of worry may not necessarily facilitate positive health behaviors and may instead reflect the need for targeted counselling, education and behavior-support interventions.

The reason for this mismatch is still unknown. It could be hypothesized that because there is currently no specific, tailored dietary intervention protocol for LS, meaningful dietary changes do not occur. Consequently, patients are left to self-navigate. While 37% of our cohort reported actively avoiding certain foods to prevent cancer, their sources of information were highly decentralized, relying primarily on the internet or their doctors rather than specialized dietetic advice.

### 4.2. Evidence for Dietary Intervention in Lynch Syndrome

For years, general population guidelines have championed broad “healthy eating” indices, such as reducing red and processed meat while increasing fiber, to lower CRC risk. Yet, several prospective cohort studies in LS populations have reported that overall general dietary quality indices do not significantly alter the development of colorectal tumors. [13,14,15] However, these null findings may stem from the specific dietary quality metrics employed, many of which lack specificity for colorectal cancer. For instance, Botma et al. derived four empirical dietary patterns from food frequency questionnaires in an LS cohort, but found a significant association with adenomas only for a pattern high in “snack” foods [12]. Notably, these patterns did not account for broader dietary confounders, failed to quantify macronutrient intake, and remain difficult to translate to other populations. Similarly, Brouwer et al. [14] utilized the Adapting Dietary Inflammatory Index (ADII), [13] a metric originally validated against a composite score of systemic inflammatory biomarkers (including IL-6, IL-8, IL-10, TNF-*α* and hs-CRP) in patients with diabetes [31]. The ADII heavily weights micronutrient intake while potentially misclassifying complex dietary habits, such as treating alcohol as anti-inflammatory, and has not demonstrated a reliable association with CRC diagnosis or recurrence in general population observational studies [32].

Furthermore, the choice of covariates in these longitudinal analyses warrants scrutiny. Eijkelboom et al. [15] evaluated the Dietary Approaches to Stop Hypertension (DASH) score [33] and the Dutch Healthy Eating Index 2015 [34], both well-associated with reduced CRC risk in the general population, but found no association within a cohort of 490 LS patients [14]. However, this study looked at a different population from ours—more than 24% of their cohort had prior colonic resection or subtotal colectomy, fundamentally anatomically changing the risk of metachronous cancer, along with potentially altering the colonic microbiome. Additionally, the hazard ratios in their study were not adjusted for BMI. Anthropometric metrics, including absolute weight status, incremental BMI increases, and adulthood weight gain, are highly significant modifiers of cancer penetrance and adenoma risk in LS [3]. Win et al. demonstrated a clear dose–response relationship in which each unit increase in BMI was associated with a 30% increase in CRC risk [35]. Interestingly, this risk was highly specific to male biological sex and the pathogenic variant of a gene. After adjustment, the relationship between higher BMI and increased cancer risk was only significant in men, perhaps due to their higher rates of metabolically active visceral adiposity than women. Moreover, the risk remained significant exclusively among MLH1 mutation carriers and combined MLH1/PMS2 carriers, but not in MSH2 or MSH6 carriers [35]. Because BMI operates as such a potent, mutation-specific driver of penetrance, omitting it from multivariate models likely introduced residual confounding, masking the true relationship between dietary patterns and colorectal outcomes in these historical LS cohorts.

### 4.3. Future Directions for Dietary Research

This pattern of data suggests that a definitive dietary target or pattern to reduce the risk of CRC in patients with LS has yet to be established, largely due to the heterogeneous dietary quality tools utilized in historical literature and a frequent failure to account for potent disease-specific risk modifiers such as BMI and specific genetic risk. While high adherence to a Mediterranean diet is associated with a 21% reduction in CRC within the general population [11] its preventive efficacy has not been prospectively evaluated in LS. Our study is only the second to investigate Mediterranean dietary patterns in an LS cohort, [19] with both existing studies being cross-sectional in nature. Specifically, we found that higher BMI continuously reduced the odds of high MEDAS adherence (aOR 0.89), while individuals who made the choice to actively restrict their diet for cancer prevention had significantly higher odds of maintaining a high Mediterranean diet score (aOR 0.33 for non-restrictors).

Patients are clearly motivated to alter their eating habits to mitigate cancer risk, but they require precise, evidence-based targets to enact behavior change. A highly promising frontier for future dietary trials may lie in shifting focus toward specific nutrients or microbiome-targeted dietary strategies. Preclinical models demonstrate that diets low in fermentable fiber promote the expansion of genotoxic *pks* + *Escherichia coli*, a microbe enriched in LS tumors that induces DNA double-strand breaks which host MMR-deficient machinery cannot effectively repair, potentially driving CRC development in LS [36,37]. Conversely, dietary fiber supplementation increases protective short-chain fatty acids (SCFAs), reduces genotoxic microflora, and halts colibactin-mediated tumorigenesis [36]. Additionally, the role of red and processed meat has not been explored in this cohort, despite their known implications for the gut microbiome and colorectal cancer development in the general population [9]. Mechanistically, high consumption of red and processed meats alters gut microbial composition, promoting pro-inflammatory metabolites such as secondary bile acids while generating DNA-damaging N-nitroso compounds and heme iron that drive colonic carcinogenesis [38]. Although gut microbiome samples were not collected in the present study, combining our behavioral insights with prospective trials that track fiber and animal protein microbiome interactions may be essential to deliver the precise, therapeutic dietary direction and research that patients with LS require.

This study has several limitations. The cross-sectional design precludes any assumptions of causality regarding the relationships between cancer worry, dietary beliefs, and actual eating habits. Additionally, our sample was drawn from a single tertiary gastrointestinal risk management clinic, which may introduce selection bias toward individuals who are already highly engaged with regular medical surveillance. Dietary intake was also self-reported via a food frequency questionnaire, introducing the potential for recall and social desirability biases, which may partially explain why participants systematically overestimated the healthiness of their diets. Dietary adherence was assessed using a 40-item FFQ with a 1-week recall window. This timeframe was specifically chosen to minimize recall bias and reduce participant burden compared to lengthy, multi-month dietary instruments. However, a short recall period may not fully capture long-term habitual variation or seasonal dietary shifts, which are relevant when benchmarking against long-term cancer prevention outcomes. To mitigate this limitation, dietary patterns were also assessed using the Mediterranean Diet Adherence Sceener. The original MEDAS, which evaluates weekly dietary intake, has been demonstrated to serve as a feasible, reproducible, and reliable tool for exploring the relationships between dietary intake and cancer development [39,40].

Conversely, a key strength of this study is the deeply characterized nature of the cohort, allowing for the concurrent evaluation of validated clinical metrics, including FFQ dietary data, exercise habits, smoking status, and measured anthropometry, within the same high-risk individuals. By directly comparing these habits against national bowel cancer guidelines, we have provided a concrete, quantifiable assessment of the behavioral gaps in modern hereditary cancer management.

## 5. Conclusions

In summary, patients with LS represent a highly worried and deeply motivated cohort. Yet, a stark paradox persists; they consume sub-optimal diets that fail to meet objective bowel cancer prevention guidelines and have poor adherence to healthy diet patterns, despite believing that they eat well. While they demonstrate exceptionally high compliance with non-dietary guidelines, such as regular cancer screening, smoking cessation, and adequate physical activity, the vast majority remain living with overweight or obesity, and their self-selected diets remain heavily saturated with established colorectal cancer risk factors. A lack of specific targeted dietary guidelines for LS due to an absence of evidence in this area may be contributing to this mismatch. Ultimately, bridging this gap through targeted clinical trials that integrate behavioral motivations with precise dietary guidelines represents a critical opportunity to empower these at-risk patients and improve long-term oncological outcomes.

## Figures and Tables

**Table 1:** Participant demographics with Lynch syndrome in a GI risk management clinic.

Demographics	Male (n = 33)	Female (n = 61)	*p*
Age	50.3 ± 12.9	51.3 ± 13.0	0.72
Country of birth *Australia* *France* *India* *Iran* *Republic of Ireland* *New Zealand* *Poland* *Sri Lanka* *UK* *USA*	27 (84%) 1 (3%) 1 (3%) 0 (0%) 1 (3%) 0 (0%) 0 (0%) 1 (3%) 0 (0%) 1 (3%)	53 (90%) 0 (0%) 0 (0%) 1 (2%) 1 (2%) 1 (2%) 2 (3%) 0 (0%) 1 (2%) 0 (0%)	0.32
Completed Education Year 12 or less TAFE or Graduate Certificate Undergraduate Degree Postgraduate Degree	4 (13%) 10 (32%) 10 (32%) 7 (23%)	10 (17%) 17 (29%) 18 (31%) 13 (22%)	0.96
Smoking status Never smoker Ex-smoker Current smoker	15 (48%) 13 (42%) 3 (10%)	37 (65%) 18 (32%) 2 (4%)	0.24
Age of diagnosis of LS	40.4 ± 13.6	42.6 ± 13.0	0.49
Impacted gene MLH1 MSH2 MSH6 PMS2 EPCAM	6 (18%) 11 (33%) 9 (27%) 6 (18%) 1 (3%)	6 (10%) 12 (20%) 29 (48%) 14 (23%) 0 (0%)	0.13
% who had previous cancer	13 (41%)	19 (32%)	0.42
Family history of malignancy Yes No Unknown/Unclear	30 (97%) 0 (0%) 1 (3%)	55 (93%) 2 (3%) 2 (3%)	0.58
BMI (kg/m^2^)	28.5 ± 4.6	27.5 ± 6.1	0.43
BMI Categories Underweight (BMI < 18.5 (kg/m^2^) Healthy weight (BMI 18.5–25 (kg/m^2^) Overweight (BMI 25–29.9kg/m^2^) Obese (BMI ≥ 30 kg/m^2^)	0 (0%) 7 (23%) 12 (39%) 12 (39%)	2 (4%) 20 (36%) 16 (29%) 18 (32%)	0.38
Aspirin use (yes)	25 (76%)	44 (72%)	0.70
% Believed had a healthy diet Yes No	21 (68%) 10 (32%)	45 (76%) 14 (24%)	0.38
MEDAS Score *	5 (4–6)	6 (4–7)	0.11
MEDAS Categories MEDAS ≤ 7 MEDAS > 7	29 (88%) 4 (12%)	51 (84%) 10 (16%)	0.58
Physical activity * Moderately active (hours/week) Vigorously active (hours/week) Strength training (days/week)	7 (5–13) 2 (0–4) 0 (0–2)	5 (3–10) 1 (0–4) 2 (0–4)	0.10 0.52 0.03
Median CWS	14 (12–15.5)	15 (12–17.5)	0.23
CWS Categories CWS ≤ 11 CWS > 11 CWS ≤ 13 CWS > 13	6 (19%) 26 (81%) 14 (44%) 18 (56%)	8 (13%) 52 (87%) 22 (37%) 38 (63%)	0.49 0.51

Data presented as mean ± standard deviation, LS: Lynch syndrome, MEDAS: Mediterranean diet score, CWS: Cancer worry score, * data presented as median and interquartile range. Numbers do not add up to column totals if individuals had not answered that specific question.

**Table 2:** Components of Mediterranean diet score in participants with Lynch syndrome.

MEDAS Components	Male	Female	*p*
Olive oil as main culinary fat	25 (78%)	49 (82%)	0.68
mL olive oil consumed/day	10 (5–15)	7.5 (5–15)	0.29
Vegetable servings 0 per day 1 serve per day 2 serves per day 3 or more serves	0 (0%) 7 (22%) 12 (38%) 13 (41%)	1 (2%) 8 (13%) 26 (43%) 25 (42%)	0.65
Fresh Fruit servings 0 per day 1 serve per day 2 serves per day 3 or more serves	6 (19%) 13 (41%) 10 (31%) 3 (9%)	7 (12%) 27 (45%) 20 (33%) 6 (10%)	0.83
Serves of red meat per day 0 ≥1	7 (22%) 25 (78%)	25 (42%) 35 (58%)	0.06
Butter/margarine serves per day 0 serves 1 serve 2 serves 3 or more serves	7 (22%) 22 (69%) 3 (9%) 0 (0%)	19 (32%) 31 (52%) 9 (15%) 1 (2%)	0.42
Sweet or carbonated beverages 0 serves ≥1 serve	20 (62%) 12 (38%)	44 (73%) 16 (27%)	0.28
Wine per week <7 glasses ≥7 glasses	29 (94%) 2 (6%)	54 (92%) 5 (8%)	0.73
Legume serves/week 0 serves 1 serve 2 serves 3 serves 4 serves or more	12 (39%) 12 (39%) 3 (10%) 2 (6%) 2 (6%)	16 (27%) 27 (46%) 9 (15%) 3 (5%) 4 (7%)	0.80
Servings of fish/shellfish per week <3 servings ≥ 3 servings	27 (87%) 4 (13%)	56 (95%) 3 (5%)	0.19
Servings of nuts/week 0 serves 1 serve 2 serves 3 serves 4 serves or more	8 (26%) 9 (29%) 8 (26%) 1 (3%) 5 (16%)	10 (17%) 19 (32%) 11 (19%) 7 (12%) 12 (20%)	0.52
Do you preferentially consume turkey/chicken/rabbit instead of veal, etc. No Yes	9 (28%) 23 (72%)	19 (32%) 41 (68%)	0.73
How many times per week do you consume vegetables, pasta, rice, or other dishes seasoned with sofrito (sauce made with tomato and onion, leek, or garlic and simmered with olive oil)? 0 1 ≥2	4 (13%) 15 (48%) 12 (39%)	17 (29%) 18 (31%) 24 (41%)	0.14

**Table 3:** Adherence to Australian colorectal cancer prevention guidelines.

	Australian Bowel Cancer Recommendations [24,25]	Male	Female	*p*
Smoking	Nil	28 (90%)	55 (96%)	0.23
Alcohol	<14 standard drinks/week	29 (94%)	57 (97%)	0.50
Processed meat	Nil	16 (50%)	44 (73%)	0.03
Whole grains	≥90 g/day	9 (30%)	13 (24%)	0.52
Unprocessed red meat	<455 g/week	23 (72%)	40 (67%)	0.61
Dairy	400 g dairy products/day	15 (47%)	34 (57%)	0.37
Aerobic exercise	30 min/day	28 (90%)	46 (78%)	0.15
BMI	<25 kg/m^2^	7 (23%)	22 (39%)	0.11
Met All Requirements		0 (0%)	1 (2%)	0.46

## Data Availability

The original contributions presented in this study are included in the article. Further inquiries can be directed to the corresponding author.
